# GMP-compatible and xeno-free cultivation of mesenchymal progenitors derived from human-induced pluripotent stem cells

**DOI:** 10.1186/s13287-018-1119-3

**Published:** 2019-01-11

**Authors:** Madison McGrath, Edmund Tam, Martina Sladkova, Athbah AlManaie, Matthew Zimmer, Giuseppe Maria de Peppo

**Affiliations:** The New York Stem Cell Foundation Research Institute, 619 West 54th Street, New York, NY 10019 USA

**Keywords:** Cell therapy, Fetal bovine serum, Good Manufacturing Practice, Human platelet lysate, Induced pluripotent stem cell, Mesenchymal stem cell, Xeno-free, Regenerative medicine

## Abstract

**Background:**

Human mesenchymal stem cells are a strong candidate for cell therapies owing to their regenerative potential, paracrine regulatory effects, and immunomodulatory activity. Yet, their scarcity, limited expansion potential, and age-associated functional decline restrict the ability to consistently manufacture large numbers of safe and therapeutically effective mesenchymal stem cells for routine clinical applications. To overcome these limitations and advance stem cell treatments using mesenchymal stem cells, researchers have recently derived mesenchymal progenitors from human-induced pluripotent stem cells. Human-induced pluripotent stem cell-derived progenitors resemble adult mesenchymal stem cells in morphology, global gene expression, surface antigen profile, and multi-differentiation potential, but unlike adult mesenchymal stem cells, it can be produced in large numbers for every patient. For therapeutic applications, however, human-induced pluripotent stem cell-derived progenitors must be produced without animal-derived components (xeno-free) and in accordance with Good Manufacturing Practice guidelines.

**Methods:**

In the present study we investigate the effects of expanding mesodermal progenitor cells derived from two human-induced pluripotent stem cell lines in xeno-free medium supplemented with human platelet lysates and in a commercial high-performance Good Manufacturing Practice-compatible medium (Unison Medium).

**Results:**

The results show that long-term culture in xeno-free and Good Manufacturing Practice-compatible media somewhat affects the morphology, expansion potential, gene expression, and cytokine profile of human-induced pluripotent stem cell-derived progenitors but supports cell viability and maintenance of a mesenchymal phenotype equally well as medium supplemented with fetal bovine serum.

**Conclusions:**

The findings support the potential to manufacture large numbers of clinical-grade human-induced pluripotent stem cell-derived mesenchymal progenitors for applications in personalized regenerative medicine.

**Graphical abstract:**

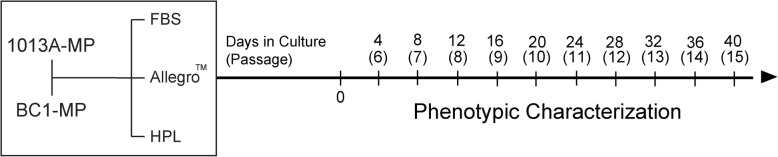

**Electronic supplementary material:**

The online version of this article (10.1186/s13287-018-1119-3) contains supplementary material, which is available to authorized users.

## Introduction

The ability to treat diseases via administration of stem cells is becoming a plausible therapeutic option [2]. Human mesenchymal stem cells (MSCs), in particular, are a promising candidate for the prevention or treatment of a large number of different medical conditions. This is because of their ability to regulate immunological and inflammatory responses and boost tissue repair and healing [35]. Human adult MSCs can be isolated from many tissues, including bone marrow, periosteum, fat, skeletal muscle, and synovial fluid, as well as from the cord blood, umbilical cord, and placenta [15]. In recent years, these cells have been used for the treatment of hematopoietic [24], cardiovascular [23], and autoimmune diseases [34] and for the repair of traumatic bone and cartilage injuries [10], with numerous clinical trials conducted worldwide every year [38]. Following administration, human MSCs do not engraft permanently but act as “drugstores” and exert their therapeutic effects via the transient release of trophic factors [9]. This implies that large numbers of cells would need to be administered to achieve therapeutic efficacy. The same is true if MSCs are to be used to engineer tissue grafts for replacement therapies [17, 30, 31]. Unfortunately, human MSCs derived from adult tissues exhibit limited proliferation potential, and therapeutically, functional cells may not be available in sufficient numbers for every patient [3, 7, 11, 37, 40].

To overcome these limitations and advance stem cell treatments, we and other research groups have recently derived mesenchymal progenitor (MP) cells from human-induced pluripotent stem cells (iPSC) [5, 16, 36]. Human iPSC-MP cells meet all criteria defining MSCs, including adherence to plastic, spindle morphology, surface expression profile, and lineage commitment. In addition, we recently demonstrated that human iPSC-MP cells display a similar expression profile for several genes involved in germ layer specification and development [16, 38] and respond in a similar fashion when subjected to specific culture conditions in vitro [16]. Unlike MSCs, iPSC-MP cells can be derived from every patient and produced in clinically sufficient numbers as a promising alternative source of functional cells for therapies. For translation into clinical practice, human iPSC-MP cells must be manufactured under xeno-free conditions and according to Good Manufacturing Practice (GMP) guidelines. In fact, while the use of animal components carries the risk of zoonoses and xenogeneic immune reactions [1], lab-made media do not undergo the quality and safety testing that is required for clinical applications. Unfortunately, there is currently no data on the behavior of human iPSC-MP cells cultured in xeno-free and GMP-compatible conditions. Previous studies using human MSCs have demonstrated that replacement of fetal bovine serum with xeno-free alternatives or use of GMP-compatible media affects cell morphology, proliferation, and phenotype but does not compromise their clinical potential as mesenchymal cells [4, 12, 25, 28]. Therefore, in this study, we explored the effects of clinically relevant media on the behavior of human iPSC-MP cells (lines 1013A and BC1). We expanded these lines for ten passages in media supplemented either with fetal bovine serum (FBS) or human platelet lysates (HPL, xeno-free) or in the commercial GMP-compatible low-serum Allegro™ Unison Medium. We then investigated the effects of long-term culture in these media on morphology, viability, mesenchymal characteristics, gene expression, and release of trophic factors.

## Materials and methods

### Cell culture

1013A-MP and BC1-MP cells were derived as previously described [16]. At P5, cells were thawed and expanded in traditional culture medium consisting of Knockout™ DMEM (Thermo Fisher Scientific, Waltham, MA), 1 ng/mL β-fibroblast growth factor (Thermo Fisher Scientific), GlutaMAX (1X; Thermo Fisher Scientific), MEM non-essential amino acids solution (1X; Thermo Fisher Scientific), 20% (*v*/*v*) HyClone™ FBS (Fisher Scientific, Pittsburgh, PA), Antibiotic-Antimycotic (1X; Thermo Fisher Scientific), and 0.1 mM β-mercaptoethanol (Gibco™, Thermo Fisher Scientific).

At confluence (85–90%), the cells were detached using trypsin/ethylenediaminetetraacetic acid (EDTA) (0.25%; Thermo Fisher Scientific), counted using a hemocytometer, and seeded at a density of 10,000 cells/cm^2^ in gelatin (0.1%; Thermo Fisher Scientific)-coated plasticware (Corning®, New York, NY). Cells were then cultured for ten passages in xeno-free medium consisting of Knockout™ DMEM (Thermo Fisher Scientific), 11.25 ng/mL heparin (Sigma-Aldrich, St. Louis, MI), 10% (*v*/*v*) human pooled platelet lysate (New York Blood Center, New York, NY), 1 ng/mL β-fibroblast growth factor, GlutaMAX (1X; Thermo Fisher Scientific), MEM non-essential amino acids (1X; Thermo Fisher Scientific), 0.1 mM β-mercaptoethanol (Gibco™, Thermo Fisher Scientific), and Antibiotic-Antimycotic (1X; Thermo Fisher Scientific) or the commercially available Allegro™ Unison hMSC Basal Medium (RoosterBio Inc., Frederick, MD) supplemented with the Allegro™ Unison hMSC Medium Supplement A (RoosterBio Inc). Traditional expansion medium supplemented with FBS (described above) was used as a control medium in all experiments. Cells were split every 4 days for the entire duration of the experiment.

Cultures were screened for mycoplasma using the MycoAlert Mycoplasma Detection Kit (Lonza, Basel, CH) following the manufacturer’s instructions.

### Expansion potential and cell viability

To study the expansion potential of 1013A-MP and BC1-MP cells cultured in different media, cells were seeded at a density of 10^4^ cells/cm^2^ in gelatin (0.1%; Thermo Fisher Scientific)-coated T25 flasks (Corning®). At the end of each passage (day 4), cells were detached using trypsin/EDTA (0.25%, Thermo Fisher Scientific) and counted using the Countess™ automated cell counter (Invitrogen). Briefly, 20 μL of single-cell suspensions was mixed with an equal volume of trypan blue stain (0.4%; Invitrogen), vortexed thoroughly, and analyzed to count live and dead cells. The proliferation potential of the cells in different culture media was expressed in terms of cumulative growth, whereas cell viability was expressed as a percentage of viable cells out of total cells.

### Immunohistochemistry

Lack of reversion to a pluripotent state during long-term culture in different media was assessed via immunohistochemistry (IHC). At passages 6, 10, and 15, the cells were detached using trypsin/EDTA (0.25%; Thermo Fisher Scientific) and counted using a hemocytometer. Cell suspensions (2 × 10^4^ cells in 200 μL DPBS) were then spun on SUPERFROST® microscope slides (Thermo Fisher Scientific) using the Cytospin 4 (Thermo Fisher Scientific). Soon after, the cells were fixed with 4% paraformaldehyde solution in DPBS (*v*/*v*) (Chem Cruz®, Santa Cruz Biotechnology, Santa Cruz, CA) for 5 min and washed three times with DPBS to remove excess fixative. Following fixation, the samples were incubated with blocking solution, consisting of 10% (*v*/*v*) normal donkey serum (Jackson Immuno Research Inc., West Grove, PA) and 0.3% (*v*/*v*) Triton™ X-100 (Sigma-Aldrich) for 30 min. The cells were then incubated overnight with the primary antibodies rabbit anti-human OCT4 (1:500; cat. no. 090023) and mouse anti-human SSEA4 (1:500; cat. no. 090006) (both from Stemgent, San Diego, CA). Donkey anti-mouse Alexa Fluor® 488 (1:1000 dilution; Thermo Fisher Scientific, cat. no. A21202) and donkey anti-rabbit Alexa Fluor® 555 (1:1000 dilution; Thermo Fisher Scientific, cat. no. A31572) were used for detection. The cell nuclei were counterstained with 4′,6-diamidine-2′-phenylindole dihydrochloride (DAPI; 1:1000 dilution) (BioVision, Milpitas, CA). Parental pluripotent stem cell lines (1013A and BC1) were used as positive controls. Negative control samples were prepared using the same methodology but omitted the primary antibodies. The samples were finally washed with DPBS and mounted using the Permount® mounting medium (Fisher Scientific) for long-term storage. Fluorescent images were taken with the Olympus IX71 epifluorescence microscope (Olympus Corporation, Shinjuku, JP).

### Flow cytometry

The expression of the mesenchymal markers CD44, CD73, and CD90 was studied via flow cytometry. At passages 7, 9, 11, 13, and 15, the cells were enzymatically detached using trypsin/EDTA (0.25%, Thermo Fisher Scientific), counted, and then filtered using a cell strainer with a mesh size of 35 μm (BD Biosciences, Franklin Lakes, NJ) to obtain single-cell suspensions. After centrifugation, the cells were re-suspended in staining buffer consisting of DPBS containing 0.5% (*v*/*v*) bovine serum albumin fraction V (Invitrogen, Waltham, MA), 100 U/mL penicillin-streptomycin (Invitrogen), 2 mM EDTA (Invitrogen), and 20 mM glucose (Sigma-Aldrich) and stained with V450-conjugated anti-CD44 (cat. no. 561292), fluorescein isothiocyanate-conjugated anti-CD73 (cat. no. 561254), and phycoerythrin-conjugated anti-CD90 (cat. no. 561970) (all from BD Bioscience, San Diego, CA). The cells were incubated on ice for 20 min in the dark, washed twice with staining buffer, and analyzed using a Thermo Fisher Scientific 4-laser Attune NxT with Autosampler. Gates were set using 1% in negative controls.

### Cell painting

Production and arrangement of cytoskeleton proteins were studied during the entire experimental period. Briefly, the cells were detached using trypsin/EDTA (0.25%; Thermo Fisher Scientific), counted using a hemocytometer, and seeded at a density of 10,000 cells/cm^2^ in gelatin (0.1%; Thermo Fisher Scientific)-coated 12-well plates (Corning) at a density of 10,000 cells/cm^2^. The day after, the cells were stained for F-actin and the type III intermediate filament protein vimentin. Upon reaching optimal density for imaging, the cells were fixed using a 4% paraformaldehyde solution in DPBS (*v*/*v*) (Chem Cruz®, Santa Cruz) as described above. Thereafter, the cells were stained with Alexa Fluor® 488 Phalloidin (1:40 in blocking solution; Thermo Fisher Scientific, cat. no. A12379) and vimentin monoclonal antibody (V9) eFluor 615 (1:500 in blocking solution; Thermo Fisher Scientific, cat. no. 42989782). The cell nuclei were counterstained with DAPI (1:1000 dilution) (BioVision). Confocal images were taken with the microscope Axiovert 200 M microscope (Zeiss, Oberkochen, GE) mounted with LSM 5 Pascal exciter using the LSM 5 Pascal software (Zeiss).

### Nanostring analysis

The expression level of select genes playing a role in pluripotency and germ layer specification was investigated simultaneously using the NanoString nCounter system (Nanostring Technologies®, Seattle, WA) (see Additional file 1: Table S1 for a list of all investigated genes). At the end of passages 6, 10, and 15, the cells were detached and counted as described above. Thus, 2.5 × 10^5^ cells were collected and lysed in RLT buffer (Qiagen, Venlo, NE). Total RNA was then extracted using the RNeasy Mini Kit (Qiagen) according to the manufacturer’s instructions and quantified with the NanoDrop 8000 (Thermo Scientific) before hybridization at 65 °C for 18 h. Data were analyzed using the nSolver™ 2.5 software. Background subtraction was performed by removing from each count the mean plus two standard deviations of negative control probes. Positive control normalization was performed using the geometric mean of targets with the highest counts. ACTB, ALAS1, CLTC, POLR2A, RPL19, and TUBB were used as reference genes. Values were transformed into the standard score (*Z*-score), and agglomerative clusters (heat map) were generated from grouped data based on Euclidean distance. To identify major transcriptional changes associated with culture in the tested media, differentially expressed genes with a fold change (FC) of at least ± 5, the baseline expression, i.e., the expression level measured before the experiment, were further analyzed and compared.

### Multiplex immunoassay

The release of trophic factors responsible for the regenerative potential, paracrine regulatory effects, and immunomodulatory activity of mesenchymal cells was investigated using the ProcartaPlex™ Multiplex Assay (Invitrogen). At the end of the culture period at P6, P10, and P15, aliquots of media were collected, vortexed, and centrifuged at 10,000×*g* for 7 min to remove the particulate. Then after, 50 μL of cell culture supernatants, controls, and standards was added to a custom kit ProcartaPlex™ plate to simultaneously assay for interleukin 6 (IL-6), interleukin 8 (IL-8), monocyte chemoattractant protein 1 (MCP-1), and vascular endothelial growth factor A (VEGF-A) using color-coded magnetic beads pre-coated with analyte-specific capture antibodies according to the manufacturer’s instructions. Following detection, the analyte contents were measured using the MAGPIX™ (Invitrogen) platform equipped with the Luminex™ Acquisition Software (Thermo Fisher Scientific). Fresh culture media were used as controls for background subtraction. Data were normalized using the total cell number per each condition and expressed as “femtogram/mL/cell.”

### Image processing and generation

Image levels and image backgrounds were processed in Adobe Photoshop CC (Adobe Systems Incorporated, San Jose, CA) to improve viewing. Images were finally cropped to optimal canvas size in Preview (Apple Inc., Cupertino, CA) and combined into figure panels using Adobe Illustrator CC (Adobe Systems Incorporated).

### Statistical analysis

Statistics was performed using the GraphPad Prism 6 version 6.0e (GraphPad Software Inc., La Jolla, CA). One-way analysis of variance (ANOVA) for multiple comparisons with Bonferroni posthoc test was used to compare the effect of different media at corresponding passage. Repeated measures ANOVA with Bonferroni correction was used to compare cells cultured in the same medium at different passages. Results are shown as means ± standard deviations. A difference between the mean values for each group was considered statistically significant when the *p* value was < 0.05.

## Results

### Cell morphology, expansion potential, and viability

**Fig. 1:**
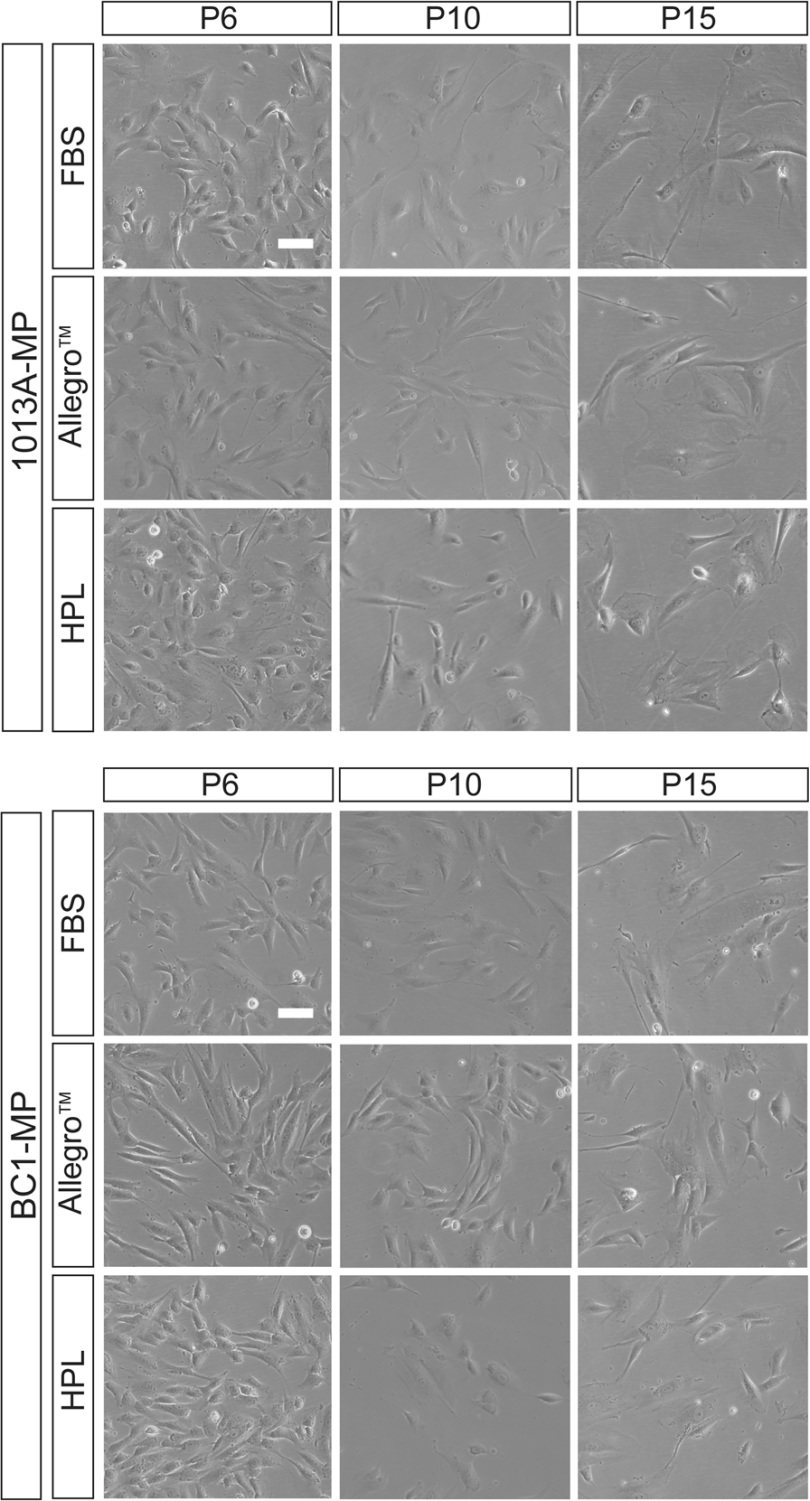
Cell morphology. Light micrographs showing the morphology of induced pluripotent stem cell-derived mesodermal progenitors (line 1013A and BC1) cultured in different media at passages 6, 10, and 15. Scale bar = 100 μm. Abbreviations: MP, mesodermal progenitors; FBS, fetal bovine serum; HPL, human platelet lysate; P, passage. Additional data are shown in Additional file 2: Figure S1

To investigate the effects of different media on cell phenotype, the morphology, expansion potential, and viability of 1013A-MP and BC1-MP cells were studied from P6 to P15. At P6, 2 days after initiating the experiment, both 1013A-MP and BC1-MP cells display a spindle-like morphology and similar size (Fig. 1) irrespective of the culture media used. Starting at P7, however, cells cultured in the Allegro™ medium begin displaying a more elongated morphology compared to cells cultured in media supplemented with either FBS or HPL, which instead preserve their initial morphology (Fig. 1 and Additional file 2: Figure S1). Besides the differences observed when comparing the different media at corresponding passage, 1013A-MP and BC1-MP cells progressively increase their size upon expansion and exhibit a more spread and flattened morphology at the end of the experiment (P15).

**Fig. 2:**
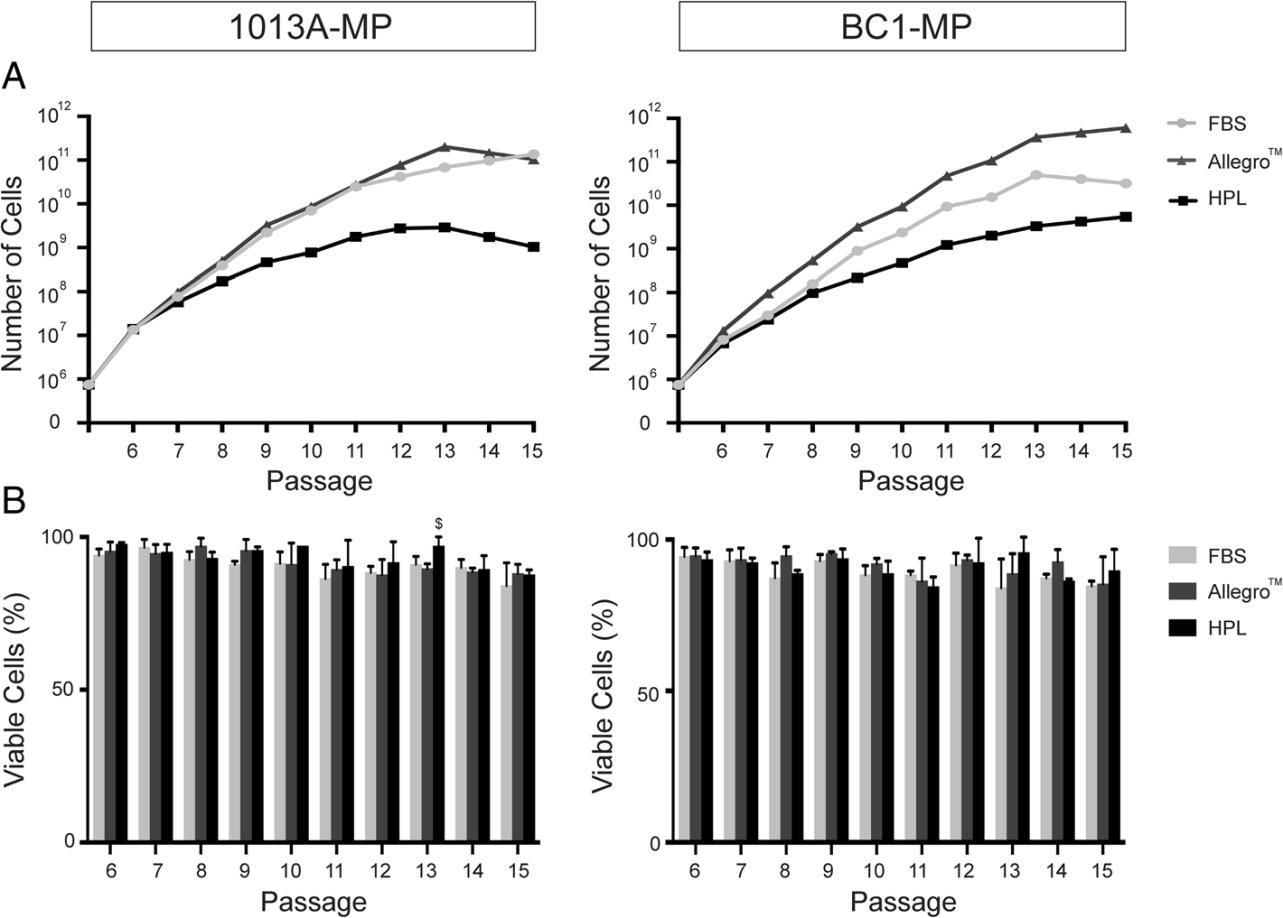
Expansion potential and cell viability. **a** Graphs showing the expansion potential of induced pluripotent stem cell-derived mesodermal progenitors (line 1013A and BC1) cultured in different media over ten passages (P6 to P15). **b** Histograms showing the viability of induced pluripotent stem cell-derived mesodermal progenitors (line 1013A and BC1) cultured in various media over ten passages (P6 to P15). Data represent averages ± SD (*n* = 3, one-way ANOVA with Bonferroni correction, *p* < .05). Abbreviations: MP, mesodermal progenitors; FBS, fetal bovine serum; HPL, human platelet lysate; P, passage

In line with the morphological changes observed, Fig. 2 shows that both 1013A-MP and BC1-MP cells grow rapidly at early passages but the expansion potential slows down later, and almost reaches a plateau at about P13-P14 in all tested media. In particular, both 1013A-MP and BC1-MP cells show higher proliferation potential when cultured in the Allegro™ medium and in the medium supplemented with FBS media compared to medium supplemented with HPL, which gives rise to about 10–100 times fewer cells than the other media. Interestingly, the ability of the different media to support cell expansion is also cell line-specific, with 1013A-MP cells growing more in the Allegro™ medium and medium supplemented with FBS and BC1-MP cells growing more in the Allegro™ medium throughout the experiment.

Despite the morphological changes and decrease in expansion potential, no significant differences in cell viability are observed between the different conditions, except when comparing 1013A-MP cells cultured in the Allegro™ medium and in medium supplemented with HPL at P13. In particular, the viability of 1013A-MP cells is ≥ 84% in medium supplemented with FBS, ≥ 88% in the Allegro™ medium, and ≥ 86.67% in medium supplemented with HPL. On the other hand, the viability of BC1-MP cells is ≥ 84% in medium supplemented with FBS, ≥ 85.33% in the Allegro™ medium, and ≥ 84.33% in medium supplemented with HPL (Fig. 2). Although no large difference is seen, the viability of 1013A-MP and BC1-MP cells seems to moderately decrease as the passage number increases.

### Pluripotency and mesenchymal markers

**Fig. 3:**
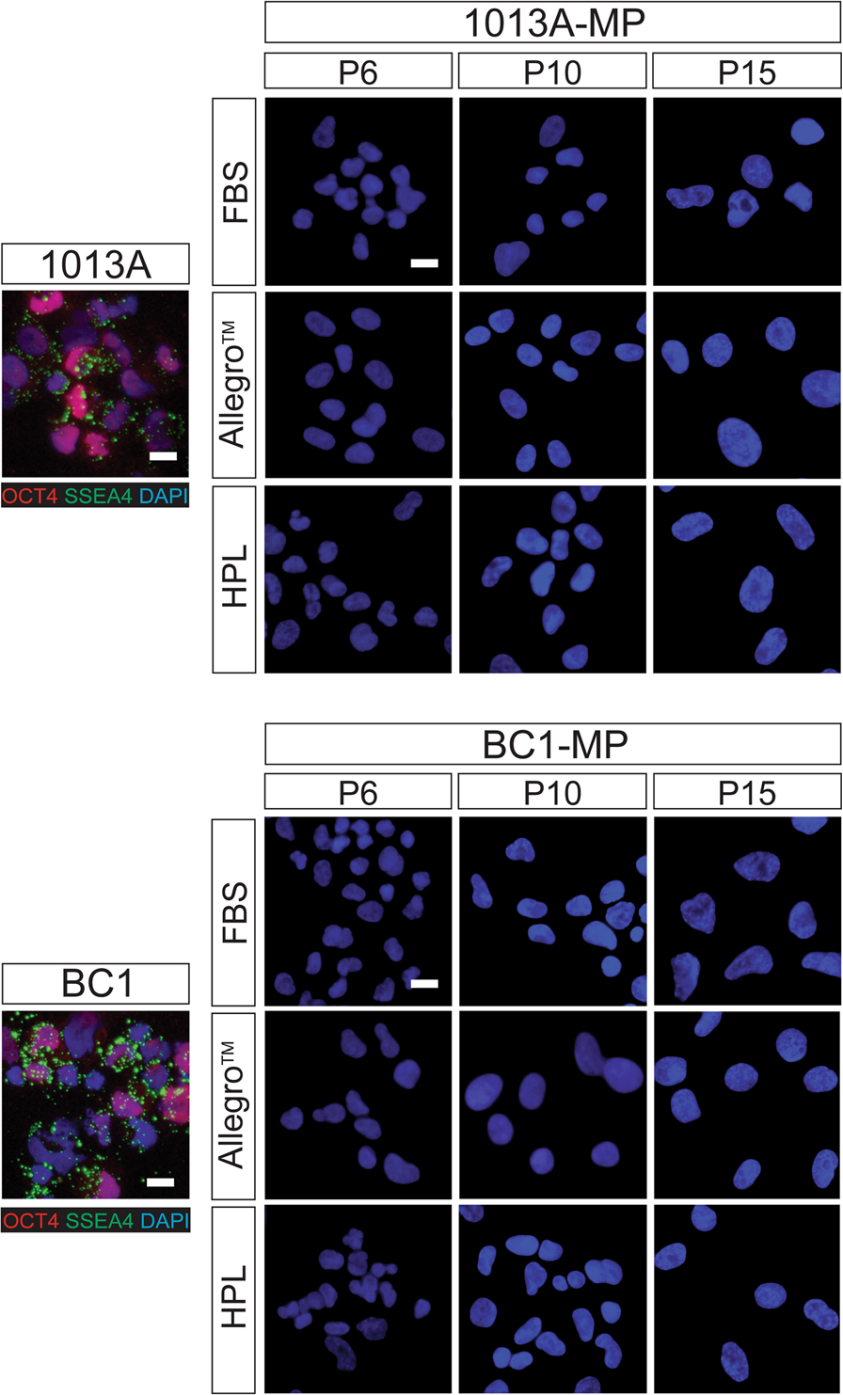
Expression of pluripotency markers. Immunohistochemical analysis showing lack of expression of pluripotency markers in induced pluripotent stem cell-derived mesodermal progenitors (line 1013A and BC1) cultured in different media at passages 6, 10, and 15. The nuclei are stained with 4′,6-diamidino-2-phenylindole (DAPI; blue). Scale bar = 20 μm. Abbreviations: MP, mesodermal progenitors; FBS, fetal bovine serum; HPL, human platelet lysate; P, passage

To verify the sustained loss of pluripotency during culture in the different media, the expression of the pluripotency markers OCT4 and SSEA4 was studied at passages 6, 10, and 15 via immunohistochemistry. Figure 3 shows that, as opposed to their parental pluripotent stem cell lines, both 1013A-MP and BC1-MP cells are negative for OCT4 and SSEA4 in all media tested and throughout the entire experiment (P6 to P15). The results also reveal that 1013A-MP and BC1-MP cells display increased nuclear size following protracted expansion in all tested media.

**Fig. 4:**
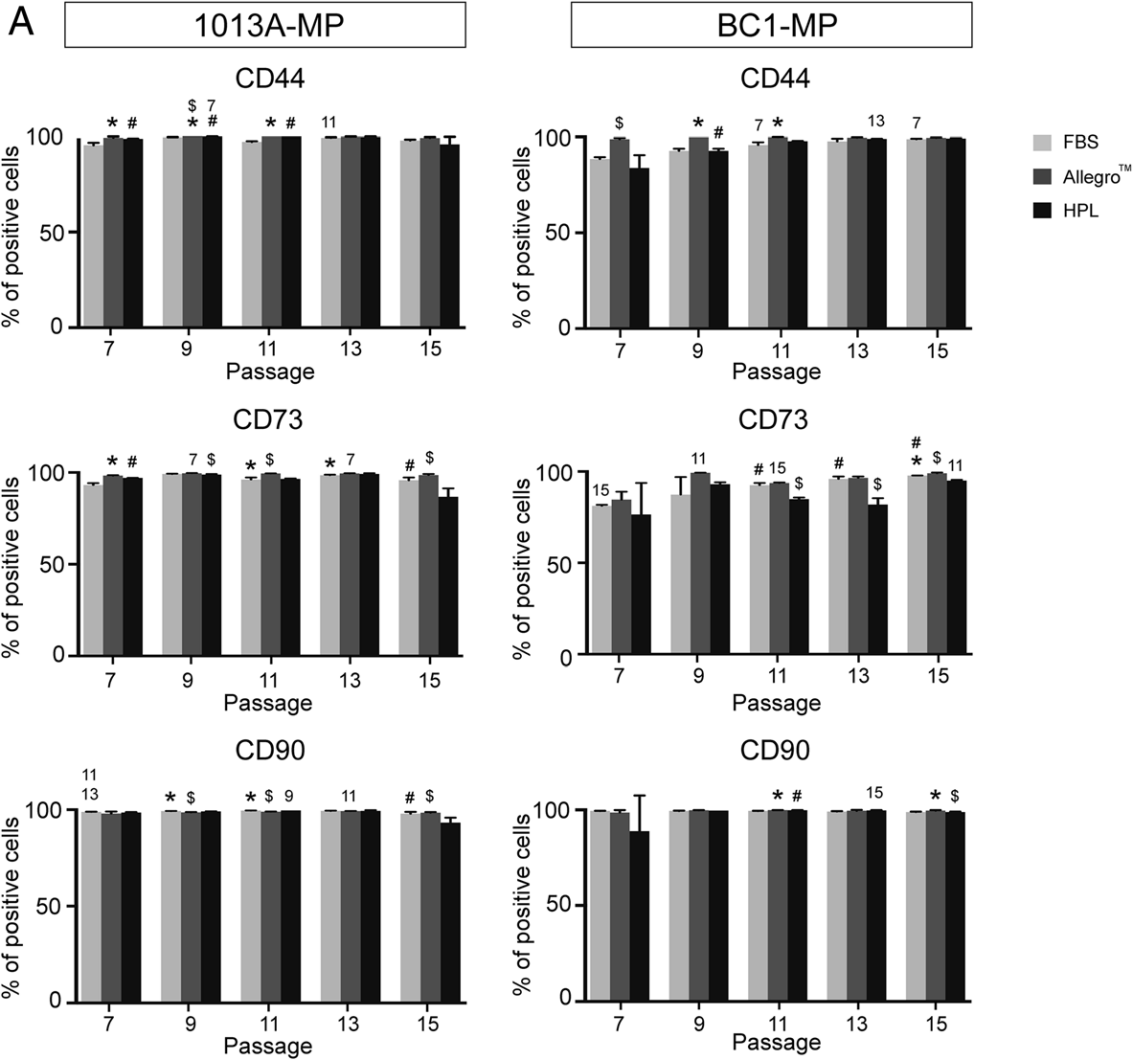
Expression of mesenchymal markers. Flow cytometry analysis showing the expression of common mesenchymal markers in induced pluripotent stem cell-derived mesodermal progenitors (line 1013A and BC1) cultured in different media at passages 7, 9, 11, 13, and 15. Data represent averages ± SD (*n* = 3, one-way ANOVA and repeated measures ANOVA with Bonferroni correction, *p* < .05). *Significant differences between FBS and Allegro™; ^#^significant differences between FBS and HPL; ^$^significant differences between Allegro™ and HPL; the numbers denote significant differences between passages in the same medium. Abbreviations: MP, mesodermal progenitors; FBS, fetal bovine serum; HPL, human platelet lysate; P, passage

To confirm the mesenchymal phenotype of the cells, the presence of common mesenchymal markers on the cell surface was analyzed via flow cytometry every other passage throughout the experiment. Figure 4 and Additional file 3: Figure S2 show that both 1013A-MP and BC1-MP cells are positive for CD44, CD73, and CD90 at all time points, irrespective of the culture medium used. Interestingly, the expression of CD44 and CD73 gradually increases upon passaging and is usually higher when the cells are cultured in the Allegro™ medium compared to medium supplemented with FBS and HPL. Specifically, the percentage of positive 1013A-MP cells varies from 84.23 to 100% for CD44, 86.75 to 99.63% for CD73, and 93.25 to 99.70% for CD90, while the percentage of positive BC1-MP cells varies from 88.53 to 99.90% for CD44, 76.43 to 99.20% for CD73, and 88.90 to 99.77% for CD90.

### Cytoskeletal proteins

**Fig. 5:**
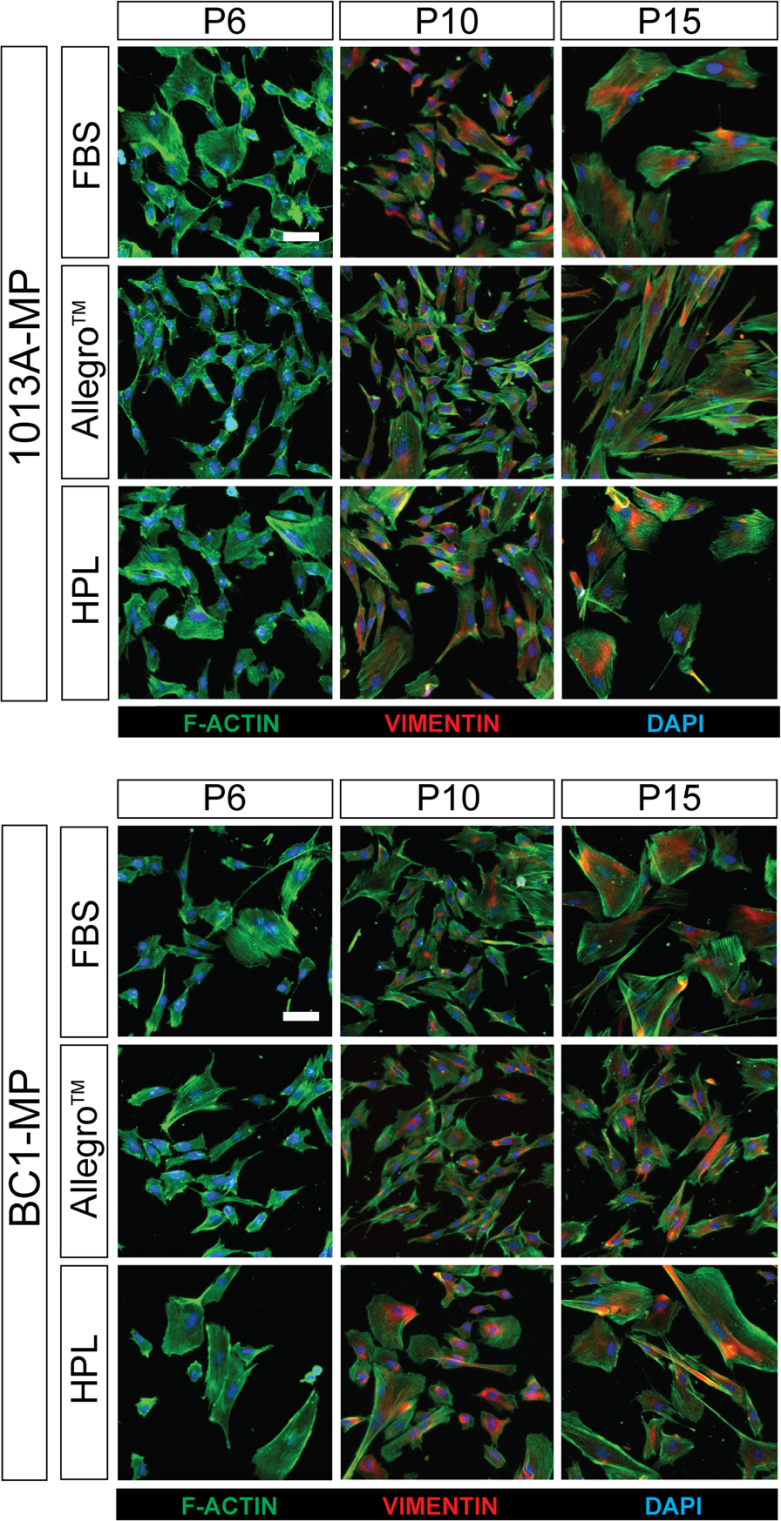
Cytoskeletal proteins. Cell painting showing the production of the cytoskeletal proteins F-actin and vimentin in induced pluripotent stem cell-derived mesodermal progenitors (line 1013A and BC1) cultured in different media at passages 6, 10, and 15. The nuclei are stained with 4′,6-diamidino-2-phenylindole (blue). Scale bar = 20 μm. Abbreviations: MP, mesodermal progenitors; FBS, fetal bovine serum; HPL, human platelet lysate; P, passage. Additional data are shown in Additional file 4: Figure S3

To study the effect of the different media on the expression and distribution of cytoskeletal proteins, F-actin and vimentin were assayed throughout the experiment (P6 to P15). The results reveal media-specific effects on the orientation of F-actin filaments, and thus on the size and morphology of the cells at corresponding passages. Figure 5 and Additional file 4: Figure S3 display representative micrographs of stained cells. In particular, the cells are round-shaped when cultured in media supplemented with either FBS or HPL, but are more spindle-like when cultured in the Allegro™ medium, especially after protracted expansion. Besides the differences in morphology, the micrographs reveal that both 1013A-MP and BC1-MP cells become larger following protracted expansion in all tested media. Interestingly, irrespective of the culture medium used, both 1013A-MP and BC1-MP cells begin to produce vimentin filaments from P8 until the end of the experimental period, with no differences apparent in the amount of vimentin filaments when the cells are cultured in the different media.

### Gene expression

**Fig. 6:**
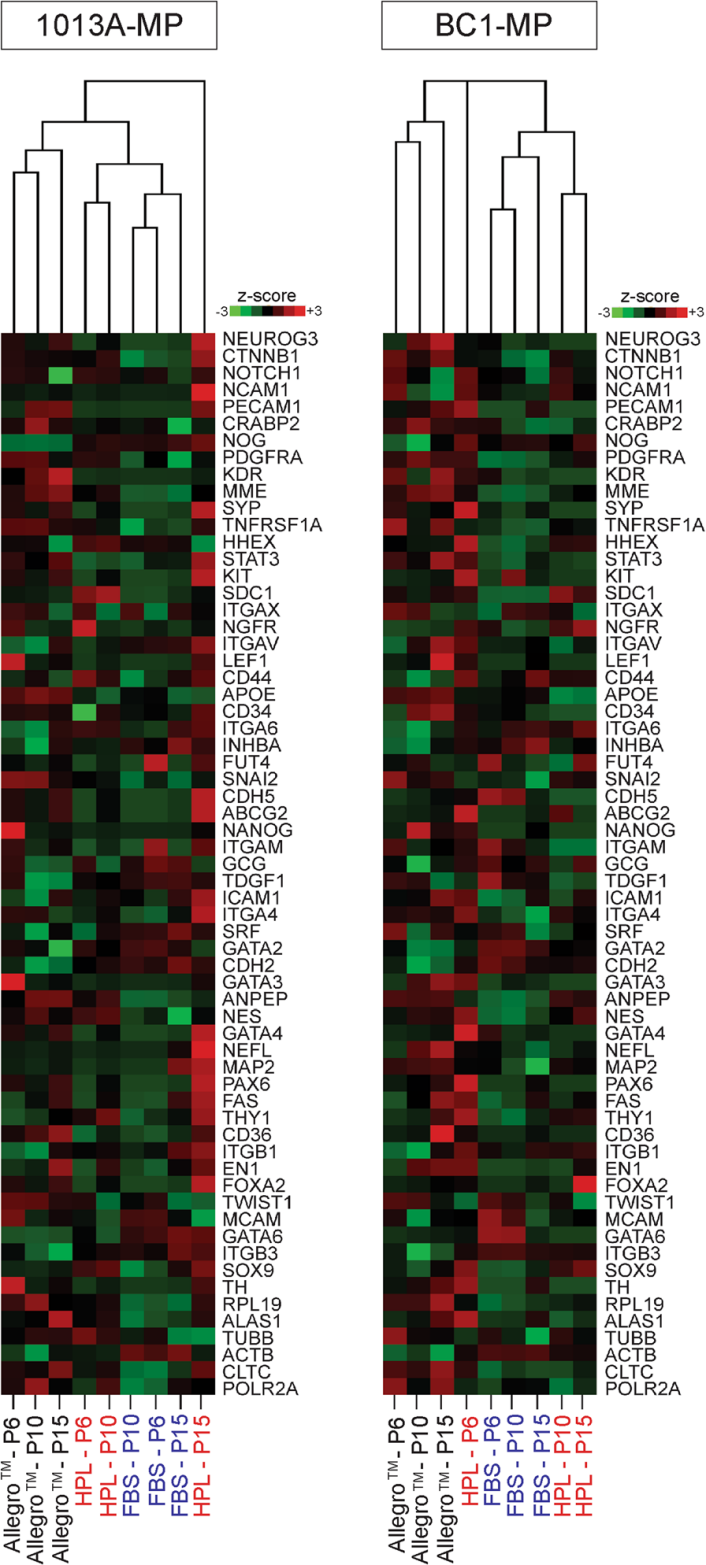
NanoString analysis. Hierarchical clustering of induced pluripotent stem cell-derived mesodermal progenitors (line 1013A and BC1) for the expression of 63 genes playing a role in pluripotency and germ layer specification. The dendograms show the expression of these genes when the cells are cultured in different media at passages 6, 10, and 15. Red represents high expression, and green represents low expression. Abbreviations: MP: mesodermal progenitors; FBS, fetal bovine serum; HPL, human platelet lysate; P, passage. Additional results are shown in Fig. 7

To gain greater insights into the molecular effects of different media on iPSC-MP cells, the expression of genes involved in pluripotency and development was studied using the NanoString technology. Out of the 88 genes analyzed (Additional file 1: Table S1), 63 are expressed in both cell lines. Importantly, 1013A-MP and BC1-MP cells do not express the pluripotency genes OCT4, NANOG, and ZFP42 throughout the experiment and in all tested media, except for 1013A-MP cells cultured in the Allegro™ medium that seem to minimally express NANOG (transcript count = 17) at P6. Hierarchical cluster analysis yields two main clusters for both 1013A-MP and BC1-MP cells, with cells cultured in media supplemented either with FBS or HPL clustering together and apart from cells cultured in the Allegro™ medium (Fig. 6). Notably, cells cultured in medium supplemented with HPL at P15 form a distinct group that sets apart from the two main clusters.

**Fig. 7:**
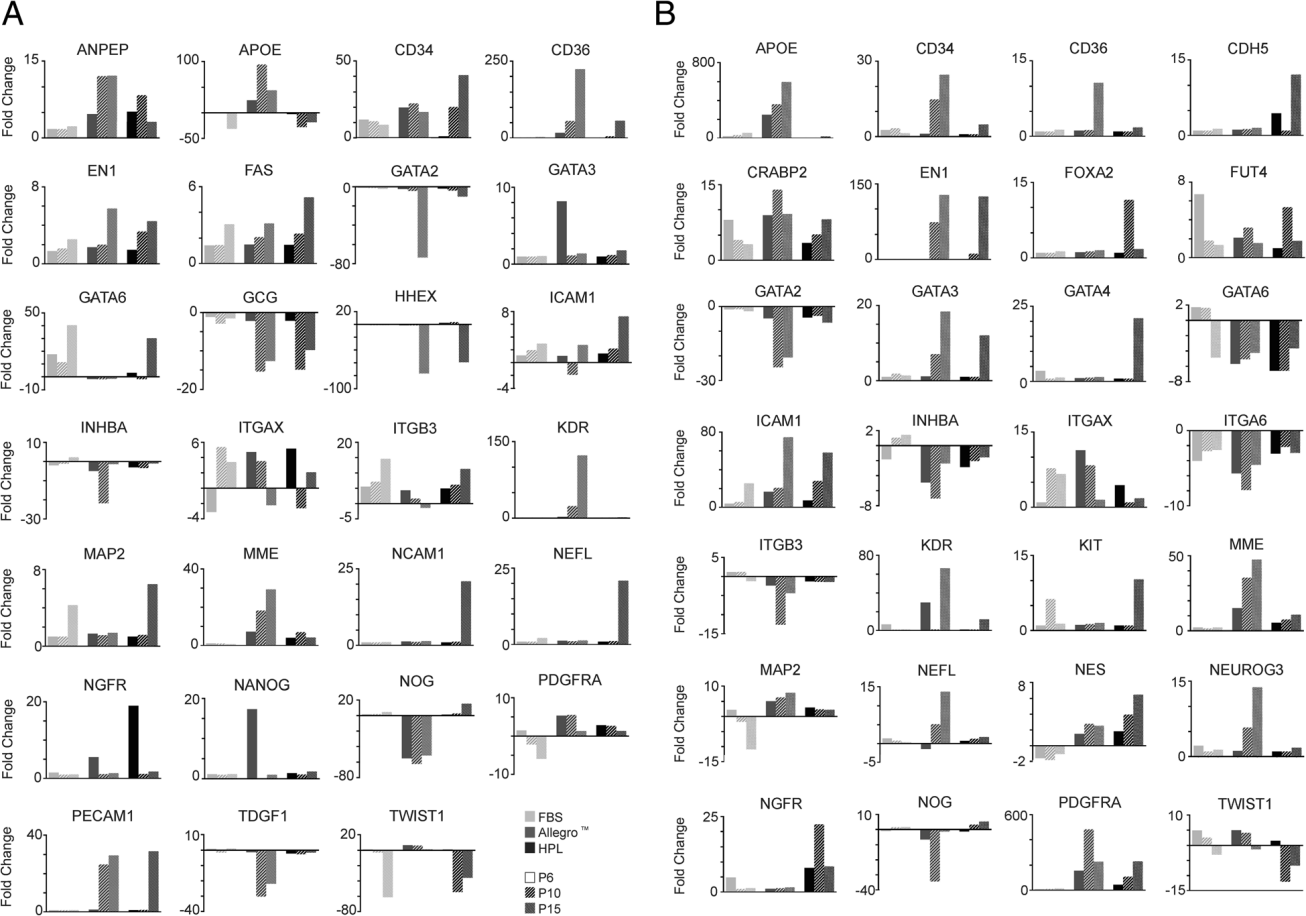
Expression of developmental genes. **a** Expression of developmental genes in induced pluripotent stem cell-derived mesodermal progenitors (line 1013A) cultured in various media at passages 6, 10, and 15. Results are shown as fold change (FC) over the expression values before the experiment. Only genes with a FC of at least ± 5 are shown. **b** Expression of developmental genes in induced pluripotent stem cell-derived mesodermal progenitors (line BC1) cultured in different media at passages 6, 10, and 15. Results are shown as FC over the expression values before the experiment. Only genes with a FC of at least ± 5 are shown. Abbreviations: MP: mesodermal progenitors; FBS, fetal bovine serum; HPL, human platelet lysate; P, passage

Analysis of differentially expressed genes, with a FC of at least ± 5 relative to baseline expression values (chosen to bring out the most pronounced differences), reveals a set of commonly regulated genes in 1013A-MP and BC1-MP cells cultured in the different media. Of the 27 (1013A-MP) and 28 (BC1-MP) identified genes with a FC of ± 5, 19 genes are common to both cell lines, and 14 display very similar transcriptional changes when the cells are cultured in the different media (Fig. 7a and b, respectively). Similarly expressed genes include APOE, CD34, CD36, EN1, GATA2, GATA3, ICAM1, INHBA, ITGAX, KDR, MME, NGFR, NOG, and TWIST.

While some transcriptional changes appear to be medium-dependent and usually associated with the culture of the cells in the Allegro™ medium, others are likely associated with protracted cell culture. The Allegro™ medium specifically affects the expression of APOE, CD36, GATA2, GATA3, INHBA, KDR, MME, NANOG, NOG, TDGF1, and TWIST in 1013A-MP cells and the expression of APOE, CD34, CD36, GATA2, GATA3, ITGB3, KDR, MME, NEFL, NEUROG3, and NOG in BC1-MP cells. On the other hand, the culture in medium supplemented with HPL specifically affects the expression of NCAM1 and NEFL in 1013A-MP cells and the expression of CDH5, FOXA2, GATA4, ITGAX, NGFR, and TWIST in BC1-MP cells. Moreover, some genes are commonly affected when the cells are cultured in the Allegro™ medium and in medium supplemented with HPL such as ANPEP, GCG, HHEX, MME, NGFR, and PDGFRA in 1013A-MP cells and GATA6, ICAM1, INHBA, MAP2, NES, and PDGFRA in BC1-MP cells. Other genes, such as EN1, FAS, and ICAM1, show transcriptional changes that are similar in all tested media and likely associated with protracted cell culture.

### Trophic factor release

**Fig. 8:**
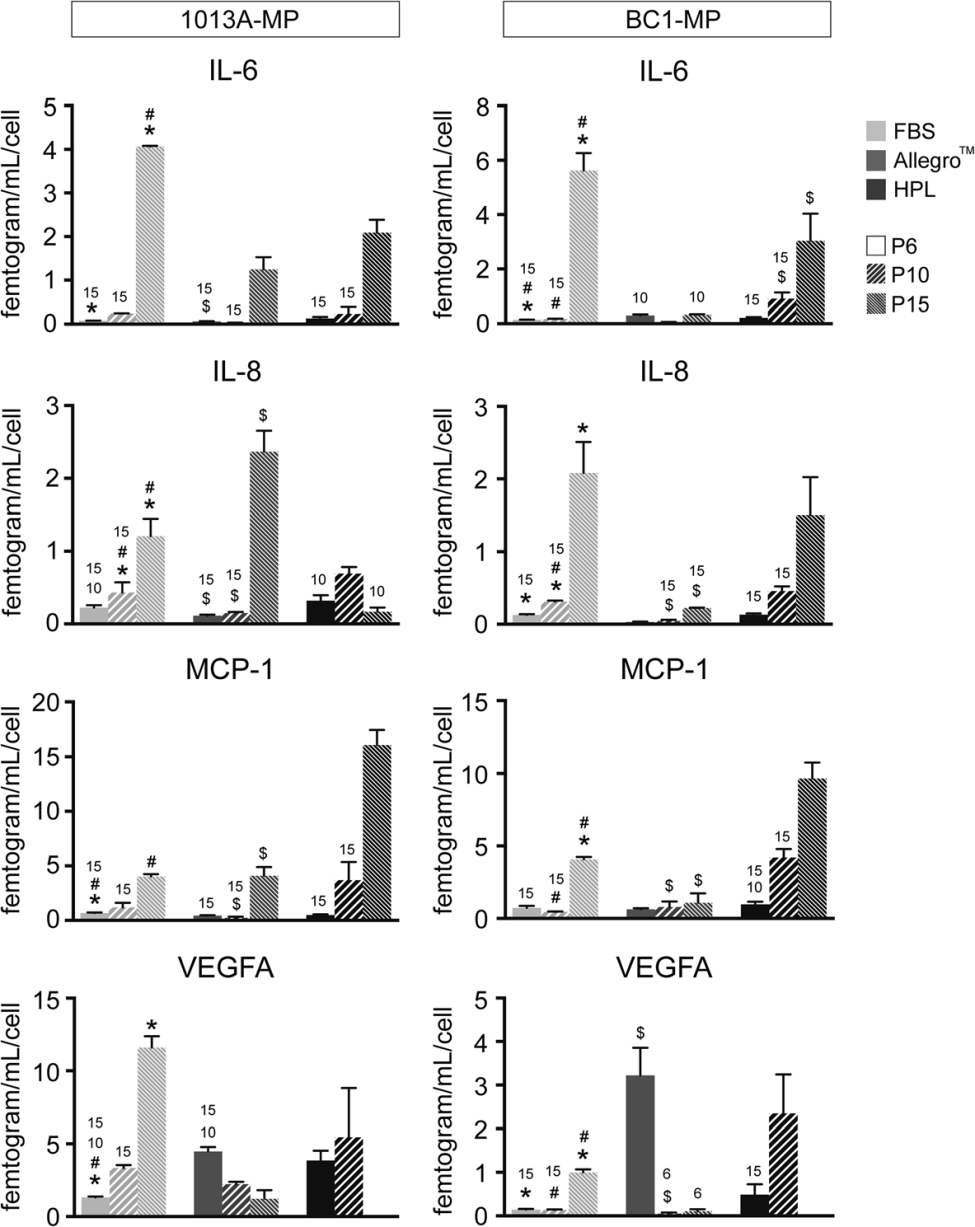
Release of trophic factors. Release of IL-6, IL-8, MCP-1, and VEGFA in induced pluripotent stem cell-derived mesodermal progenitor (line 1013A and BC1) cultured in various media at passages 6, 10, and 15. Data represent averages ± SD (*n* = 3, one-way ANOVA with Bonferroni correction, *p* < .05). *Significant differences between FBS and Allegro™; ^#^significant differences between FBS and HPL; ^$^significant differences between Allegro™ and HPL; the numbers denote significant differences between passages in the same medium. Abbreviations: FBS, fetal bovine serum; HPL, human platelet lysate; P, passage

To assess the paracrine regulatory properties of 1013A-MP and BC1-MP cells cultured in different media, the release of trophic factors was studied at P6, P10, and P15 using a multiplex immunoassay. The analysis shows that both cell types release IL-6, IL-8, MCP-1, and VEGFA with a similar pattern but in different amounts (Fig. 8). Interestingly, the release of IL-6, IL-8, and MCP-1 gradually increases upon passaging both in 1013A-MP and BC1-MP cells in all tested media, and it is significantly higher at P15 compared to earlier passages. On the other hand, the release of VEGFA increases upon passaging in medium supplemented with FBS and HPL but decreases when cells are cultured in the Allegro™ medium. Furthermore, no VEGFA release is observed at the end of the experiment (P15) when 1013A-MP and BC1-MP cells are cultured in medium supplemented with HPL.

## Discussion

MSCs reside in many human tissues and can regulate inflammation, immune response, healing, and regeneration [9, 10, 23, 24, 34, 35]. As a result of this, they have been extensively used in preclinical disease models and early phase clinical trials with promising results [38], though no FDA-approved indications exist yet. Unfortunately, MSCs are fairly scarce in adult tissues and display limited expansion potential in vitro, such that they may not be available in sufficient numbers to achieve therapeutic efficacy [21, 39]. On the other hand, human iPSC-MP cells can be generated to virtually unlimited numbers from every patient (Villa-Diaz, 2013; [5, 16]), and thus display high potential for future clinical applications.

In this study, we explored the possibility to culture and expand human iPSC-MP cells in xeno-free medium (supplemented with HPL) or using a commercial low-serum medium (Allegro™ Unison) that is GMP-compatible and can boost the expansion of human MSCs with the minimal medium exchange, thus reducing processing time and labor. Human iPSC-MP cells were cultured for 10 weeks in the different media, and the long-term effects of these conditions on cell morphology, expansion potential, viability, and phenotype were explored and compared.

The results showed that culture of human iPSC-MP cells in these media yields cells with very similar properties to those cultured in traditional medium supplemented with FBS, with the exception of improved viability as well as some changes in cell morphology, expansion potential, gene expression, and release of trophic factors. When cultured in the Allegro™ medium, the cells assume a more elongated morphology than in media supplemented with either FBS or HPL, which instead display a rounder morphology.

Interestingly, the expansion potential of both 1013A-MP and BC1-MP cells is influenced by the type of medium, with cells cultured in the Allegro™ medium and in the medium supplemented with FBS giving rise to a higher number of cells than medium supplemented with HPL. These results suggest that factors that are present in the different media either affect the cell senescence process or support enrichment of populations of more primitive cells with higher regenerative potential. Additional studies aimed at testing the clonogenic potential and functional properties of different cells present within the same cell population will help to answer these questions. In contrast with our findings, other groups have demonstrated that culture of human MSCs in medium supplemented with HPL results in a higher number of cells compared to medium supplemented with FBS [4, 20]. The discrepancy observed in the results possibly arises from the different concentrations of the supplements used, the batch-to-batch variability in FBS and HPL products, the type of cells employed, or a combination of these factors [25, 26]. For example, in order to comply with the producer’s recommendations, the amounts of HPL and FBS used in this study were 10% and 20%, respectively. This might explain the difference in expansion potential observed between these two conditions. Further studies aimed at culturing the cells using identical amounts of FBS and HPL are needed to verify this discrepancy.

The longitudinal nature of the study enabled us to discern additional properties of human iPSC-MP cells related to the duration of culture. While at early passages rapidly dividing cells give rise to a nearly homogenous population of small round cells (a process described as rounding up [13]), at later passages, cells seem to undergo dissimilar dynamics of cell division and linger in different phases of the cell cycle (mitosis or interphase), yielding a more heterogeneous population characterized by round tiny cells (mitosis) and large flattened cells (interphase) [13].

While cells are tiny and slender at early passages, they tend to become larger and plumper after prolonged culture in all tested media. At late passages, the fraction of large cells increases in all cultures, suggesting that more and more cells are reaching replicative senescence [8]. In line with a general increase in cell size associated with prolonged cell culture, in all tested media, 1013A-MP and BC1-MP cells progressively display increased nuclear size, which is also indicative of cell senescence [32]. Concomitant with the morphological changes observed, proliferation studies showed that the cells exhibit a log phase of exponential growth during the first passages but seem to reach a growth plateau state at the end of the culture period.

Phenotypic stability and lack of reversion to a pluripotent state is a *condicio sine qua non* for clinical applications of progenitors and lineage-specific cells derived from human iPSCs [33]. In this study, both cell lines did not express the pluripotent genes OCT4, NANOG, ZFP42, and SSEA4, except for 1013A-MP cells at P6 in the Allegro™ medium that seemed to minimally express NANOG. The scientific relevance of NANOG expression soon after plating 1013A-MP cells in the Allegro™ medium remains elusive and requires further investigation. On the other hand, the cells maintained a mesenchymal phenotype as evidenced by the expression of the mesenchymal markers CD44, CD73, and CD90, suggesting that the cells may still retain important functional properties following large-scale expansion in vitro. In addition to expressing typical mesenchymal markers, both 1013A-MP and BC1-MP cells stained positive for vimentin, a type III intermediate filament (IF) protein that is expressed in mesenchymal cells is responsible for maintaining the cell shape, integrity of the cytoplasm, and stabilizing cytoskeletal interactions [22]. Interestingly, the results showed that both cell lines, irrespective of the media tested, began to stain positive for vimentin after the first two passages in culture, specifically at P8. This indicates that the cells might not be fully mature at early passages following derivation and need to undergo several cell divisions before becoming developmentally equivalent to human MSCs derived from adult tissues. Previous research comparing mesenchymal progenitors derived from human pluripotent stem cells with human MSCs derived from adult tissues have supported this possibility [18, 19]. Functional studies of cells at early and late passages are needed to better validate this phenomenon and to clarify the relationship between vimentin expression and mesenchymal properties of human iPSC-MP cells.

In addition to the effects observed on cell morphology and proliferation, long-term culture of 1013A-MP and BC1-MP cells in different media affected the expression of genes involved in germ layer differentiation [6]. In order to identify major transcriptional changes associated with culture in these media, differentially expressed genes with a FC of at least ± 5, the expression level measured before the experiment, were further analyzed. The data revealed that many genes were commonly regulated when 1013A-MP and BC1-MP cells were cultured in the Allegro™ medium and in the medium supplemented with HPL, with the Allegro™ medium resulting in larger gene expression variations. Interestingly, many genes upregulated when cells were cultured in the Allegro™ medium and in the medium supplemented with HPL, or upon protracted expansion in all tested media, included genes recognized to play a role in mesodermal specification [6], suggesting that protracted cell culture in these conditions could boost the mesenchymal potential of iPSC-MP cells. The effects of these transcriptional changes on the phenotypic and therapeutic properties of human iPSC-MP cells remain to be carefully investigated before clinical translation. The presence of factors and other signaling molecules in the different media must account for the observed transcriptional changes, which involve genes encoding for adhesion molecules, cell-to-cell interaction proteins, and transcription factors. While the changes in the expression of adhesion molecules might explain the difference observed in cell morphology, changes in the expression of transcription factors could be responsible for the differences in expansion potential observed. In addition to the transcriptional changes associated with the culture of the cells in the different media, EN1, FAS, and ICAM1 displayed similar transcriptional changes during protracted expansion and are expected to be associated with the replicative senescence of the cells [27, 29].

The ability of human MSCs to regulate immunological and inflammatory responses, and boost tissue repair and healing, is associated with the release of specific trophic factors, including cytokines, chemokines, and growth factors [9]. In this study, both 1013A-MP and BC1-MP cells released IL-6, IL-8, MCP-1, and VEGFA irrespective of the tested medium. These factors exert angiogenic and other trophic effects and are thought to be responsible for the therapeutic properties of human MSCs [14]. While no large differences were observed when comparing media supplemented with FBS or HPL, cell cultured in the Allegro™ medium released lower amounts of these factors at most time points during the experiment. It is plausible that the composition and lower content of supplement in the Allegro™ medium compared to the other media are responsible for this observation. Notably, the release of these factors increased following expansion in vitro reaching highest levels at P15. As discussed above, iPSC-MP cells might be too primitive at early passages following derivation, and become more functionally mature over time, supporting the idea that iPSC-MP cells could retain important functional properties following large-scale expansion in vitro. However, further testing is necessary to fully assess the effects of these factors in regulating cell response and tissue repair.

Altogether, the results of this study show that expansion of iPSC-MP cells in GMP-compatible and xeno-free media is effective, though it results in the alterations of cell morphology, in the regulation of some genes involved in germ layer specification, and in the release of trophic factors. Many of these effects are common to both 1013A-MP and BC1-MP cells, meaning they might influence cell phenotype and therapeutic properties, although studies with human MSCs isolated from adult tissues do not support this hypothesis [20]. Additional work, both in vitro and in vivo, is needed to fully understand the effects of different culture media on the biological and paracrine regulatory properties of human iPSC-MP cells.

## Conclusions

The ability to manufacture large amounts of clinical-grade mesenchymal cells is paramount to the success of stem cell therapy using these cells. We demonstrate here that alternative culture conditions, which meet some of the requirements for clinical translation, can be employed for the large-scale expansion of human mesenchymal progenitors generated via reprogramming of somatic cells. Although additional studies are required to comprehensively understand the effects of different culture media on the properties of human iPSC-MP cells, this study paves the way towards the utilization of these cells in clinical settings for personalized stem cell treatments.

## Additional files

Additional file 1: **Table S1.** List of developmental genes analyzed using Nanostring technology. (DOCX 86 kb)

Additional file 2: **Figure S1.** Additional data on cell morphology. Light micrographs showing the morphology of induced pluripotent stem cell-derived mesodermal progenitors (line 1013A and BC1) cultured in different media at passages 7, 8, 9, 11, 12, 13, and 14. Scale bar = 100 μm. Abbreviations: MP, mesodermal progenitors; FBS, fetal bovine serum; HPL, human platelet lysate; P, passage. (TIF 69196 kb)

Additional file 3: **Figure S2.** Additional data on flow cytometry. (A) Expression of CD44, CD73, and CD90 in induced pluripotent stem cell-derived mesodermal progenitors (line 1013A) cultured in the Allegro™ medium at each passage (top row) and in all tested media at P15 (bottom row). (B) Expression of CD44, CD73, and CD90 in induced pluripotent stem cell-derived mesodermal progenitors (line BC1) cultured in the Allegro™ medium at each passage (top row) and in all tested media at P15 (bottom row). Abbreviations: FBS, fetal bovine serum; HPL, human platelet lysate; P, passage. (TIF 13140 kb)

Additional file 4: **Figure S3.** Additional data on cytoskeletal proteins. Cell painting showing the production of cytoskeletal proteins F-actin and vimentin in induced pluripotent stem cell-derived mesodermal progenitors (line 1013A and BC1) cultured in different media at passages 7, 8, 9, 11, 12, and 14. Nuclei are stained with 4′,6-diamidino-2-phenylindole (blue). Scale bar = 20 μm. Abbreviations: MP, mesodermal progenitors; FBS, fetal bovine serum; HPL, human platelet lysate; P, passage. (TIF 102410 kb)
