# Supplementary material for: GMP-compatible and xeno-free cultivation of mesenchymal progenitors derived from human-induced pluripotent stem cells
Source: Stem Cell Res Ther. 2019 Jan 11;10:11. doi: 10.1186/s13287-018-1119-3 (PMC6329105; doi:10.1186/s13287-018-1119-3)
Supplement: Supplementary file 1 — Table S1. List of developmental genes analyzed using Nanostring technology. (DOCX 86 kb) [file 13287_2018_1119_MOESM1_ESM.docx]

**Table S1: List of developmental genes analyzed using Nanostring technology**

| GENE | DESCRIPTION |
| --- | --- |
| *ABCG2* | ATP Binding Cassette Subfamily G Member 2 |
| *ADIPOQ* | Adiponectin, C1Q And Collagen Domain Containing |
| *ANPEP* | Alanyl Aminopeptidase, Membrane |
| *APOE* | Apolipoprotein E |
| *CD34* | CD34 Molecule |
| *CD36* | CD36 Molecule |
| *CD4* | CD4 Molecule |
| *CD44* | CD44 Molecule |
| *CDH1* | Cadherin 1 |
| *CDH2* | Cadherin 2 |
| *CDH5* | Cadherin 5 |
| *CDX2* | Cyclin-Dependent Kinase 2 |
| *CEACAM1* | Carcinoembryonic Antigen Related Cell Adhesion Molecule 1 |
| *CRABP2* | Cellular Retinoic Acid Binding Protein 2 |
| *CTNNB1* | Catenin Beta 1 |
| *DLL1* | Delta-Like 1 |
| *EN1* | Engrailed Homeobox 1 |
| *FAS* | Fas Cell Surface Death Receptor |
| *FGFR2* | Fibroblast Growth Factor Receptor 2 |
| *FOXA2* | Forkhead Box A2 |
| *FUT4* | Fucosyltransferase 4 |
| *GATA2* | GATA Binding Protein 2 |
| *GATA3* | GATA Binding Protein 3 |
| *GATA4* | GATA Binding Protein 4 |
| *GATA6* | GATA Binding Protein 6 |
| *GCG* | Glucagon |
| *HAND1* | Heart And Neural Crest Derivatives Expressed 1 |
| *HHEX* | Hematopoietically Expressed Homeobox |
| *HNF1A* | HNF1 Homeobox A |
| *HNF1B* | HNF1 Homeobox B |
| *ICAM1* | Intercellular Adhesion Molecule 1 |
| *INHBA* | Inhibin Beta A |
| *ISL1* | ISL LIM Homeobox 1 |
| *ITGA4* | Integrin Subunit Alpha 4 |
| *ITGA6* | Integrin Subunit Alpha 6 |
| *ITGAL* | Integrin Subunit Alpha L |
| *ITGAM* | Integrin Subunit Alpha M |
| *ITGAV* | Integrin Subunit Alpha V |
| *ITGAX* | Integrin Subunit Alpha X |
| *ITGB1* | Integrin Subunit Beta 1 |
| *ITGB3* | Integrin Subunit Beta 3 |
| *KDR* | Kinase Insert Domain Receptor |
| *KIT* | Proto-Oncogene Receptor Tyrosine Kinase |
| *LEF1* | Lymphoid Enhancer Binding Factor 1 |
| *MAP2* | Microtubule Associated Protein 2 |
| *MAPT* | Microtubule Associated Protein Tau |
| *MCAM* | Melanoma Cell Adhesion Molecule |
| *MME* | Membrane Metallo-Endopeptidase |
| *MNX1* | Motor Neuron And Pancreas Homeobox 1 |
| *MYOD1* | Myogenic Differentiation 1 |
| *MYOG* | Myogenin (Myogenic Factor 4) |
| *NANOG* | Nanog Homeobox |
| *NCAM1* | Neural Cell Adhesion Molecule 1 |
| *NEFL* | Neurofilament, Light Polypeptide |
| *NES* | Exportin 1 |
| *NEUROG3* | Neurogenin 3 |
| *NGFR* | Nerve Growth Factor Receptor |
| *NKX2-5* | NK2 Homeobox 5 |
| *NOG* | Noggin |
| *NOTCH1* | Notch 1 |
| *OTX2* | Orthodenticle Homeobox 2 |
| *PAX3* | Paired Box 3 |
| *PAX6* | Paired Box 6 |
| *PAX7* | Paired Box 7 |
| *PDGFRA* | Platelet Derived Growth Factor Receptor Alpha |
| *PDX1* | Pancreatic And Duodenal Homeobox 1 |
| *PECAM1* | Platelet And Endothelial Cell Adhesion Molecule 1 |
| *POU5F1* | POU Class 5 Homeobox 1 |
| *SDC1* | Syndecan 1 |
| *SLC2A2* | Solute Carrier Family 2 Member 2 |
| *SNAI2* | Snail Family Zinc Finger 2 |
| *SOX10* | Sex Determining Region Y-Box 10 |
| *SOX2* | Sex Determining Region Y-Box 2 |
| *SOX9* | Sex Determining Region Y-Box 9 |
| *SPI1* | Spi-1 Proto-Oncogene |
| *SRF* | Serum Response Factor |
| *SRY* | Sex Determining Region Y |
| *SST* | Somatostatin |
| *STAT3* | Signal Transducer And Activator Of Transcription 3 |
| *SYP* | Synaptophysin |
| *T* | T Brachyury Transcription Factor |
| *TDGF1* | Teratocarcinoma-Derived Growth Factor 1 |
| *TH* | Tyrosine Hydroxylase |
| *THY1* | Thy-1 Cell Surface Antigen |
| *TNFRSF1A* | Tumor Necrosis Factor Receptor Superfamily Member 1A |
| *TWIST1* | Twist Family BHLH Transcription Factor 1 |
| *XIST* | X Inactive Specific Transcript |
| *ZFP42* | Zinc Finger Protein 42 |
